# Clinicopathological significance of CD133 and CD44 expression in infiltrating ductal carcinoma and their relationship to angiogenesis

**DOI:** 10.1186/s12957-015-0486-9

**Published:** 2015-02-15

**Authors:** Zhengquan Han, Zhendong Chen, Rongsheng Zheng, Zenong Cheng, Xiaomeng Gong, Danna Wang

**Affiliations:** Department of Medical Oncology, The Second Affiliated Hospital of Anhui Medical University, No. 687, Furong Road, Hefei, 230000 Anhui Province China; Department of Medical Oncology, The First Affiliated Hospital of Bengbu Medical College, Bengbu, 233000 Anhui Province China; Department of Pathology, The First Affiliated Hospital of Bengbu Medical College of Bengbu Medical College, No. 287 Changhuai Road, Bengbu, 233000 Anhui Province China

## Abstract

**Background:**

Breast cancer is the leading cause of cancer death in females worldwide, and the majority type is infiltrating ductal carcinoma (IDC). Most of IDC patients died of metastasis and recurrence. Cancer stem cells (CSCs) are defined with the ability to be self-renewal and potentially promote proliferation and formation of tumors. CSCs are related to angiogenesis and are important targets in new cancer treatment strategies. In this study, we purposed to investigate on expression and clinical significances of CSCs marked by CD133 and CD44 in IDC and their relationship to angiogenesis.

**Methods:**

The specimens of IDC from 325 Chinese patients with follow-up were analyzed for CD133, CD44, CD82, and CD34 protein expression by immunohistochemical staining. The Pearson chi-square test and *t* test were used to assess the associations among the positive staining of these markers and clinicopathological characteristics. Postoperative overall survival time in these patients with IDC was analyzed by univariate and multivariate analyses.

**Results:**

In IDC tissues, positive rates of 48.6%, 53.8%, and 42.2% were obtained for CD133, CD44, and CD82 protein, respectively; the mean score of microvessel density (MVD) was 20.5 ± 7.0 in IDC group. And there was a significant difference between the two groups. There was a positive relationship between the expression of CD133, CD44, and the score of MVD and the grades of tumor, lymph node metastasis, tumor-node-metastasis (TNM) stages (all *P* < 0.05); and the expression of CD82 was negatively related to grades of tumor, lymph node metastasis, and TNM stages (all *P* < 0.05). The overall mean survival time of the patients with CD133, CD44, and the score of MVD (≥21) positive expression was lower than that of patients with negative expression. The overall mean survival time of patients of CD82-positive expression was longer than that of patients of the negative expression group. The positive expression of CD133 and CD82, and TNM stages were independent prognostic factors of IDC (*P* < 0.05).

**Conclusions:**

CSCs, angiogenesis, and aberrant expression of CD82 may be involved in the initiation, development, metastasis, and recurrence. It is suggested that CSCs, angiogenesis, and CD82 be possible as a therapeutic marker for anti-tumor therapy.

## Background

Caner is the leading cause of death in China. Breast cancer is the first commonly diagnosed cancer and the first cause of cancer death in women [1,2] and accounting for 23% of all malignancies [1]. It is well known that the expression profiles of estrogen receptor (ER), progesterone receptor (PR), and human epidermal growth factor receptor 2 (Her-2)/neu are closely associated with breast cancer and used for predicting the prognosis and therapy [3]. Over the past 25 years, the breast cancer death rates have been decreasing in some European and North American countries [4]. Although rapid progress has recently been made in treatment, such as China, the prognosis for patients with breast cancer remains unsatisfactory, and mortality rate of breast cancer is rising. The main reasons are metastasis, recurrence, and resistance-therapy. Consequently, it is urgent to discovery novel biomarkers predicted the diagnosis and development of breast cancer [5-7]. The infiltrating breast carcinoma is the first most frequent histological subtype of breast cancer.

Cancer stem cells (CSCs) are defined as a small population of tumor cells and termed as self-renewal, proliferation, and therapy resistance and are responsible for tumor metastasis and tumorigenicity [8-12]. CD133, which is also known as prominin-1, is a very common CSC marker and was initially considered as a marker of hematopoietic stem cells [13]. Later, CD133 was found expressing in many solid tumors, and it may represent a putative cancer stem cell marker. Additionally, CD133 has also been found to be a prognostic factor of some tumors, such as breast, colon, and lung cancer [14-16]. CD44 is a cell-adhesion molecule and is also considered as a marker of CSC. CD44 is involved in cell to cell and cell to matrix interactions and signal transduction [17]. CD44 is a complex family of molecules, which is encoded by a gene, including 20 exons. The standard CD44 (CD44s) is consisted of exons 1 to 5 and 15 to 20. Exons 6 to 15 are variable, and variable exons are commonly identified as v1 to v10, respectively. So, there are various isoforms of CD44. The role of these isoforms is not fully clear, although some are believed that they are necessary to multiple biological functions of normal cells and also mediate a critical step in some cancer metastasis [18,19]. CD44 has also been found to be a poor prognosis in many solid tumors, including liver, gastric, colon, breast, and lung cancer.

Cancer invasion and metastasis require multiple complex steps and involve a variety of molecules. Angiogenesis, which can supply blood and nutrient for cancers growth, is an important step in cancer invasion and metastasis. Microvessel density (MVD) is a common standard method of measuring cancer angiogenesis. High score of MVD in tumors means not only an aggressive phenotype of tumors but also an easy metastasis of tumor. And high score of MVD often indicates a poor prognosis [20,21]. Aberrant expression of suppressor of metastasis of tumor also plays an important role in cancer metastasis. CD82 gene is a suppressor of metastasis of tumor in many tumors and a member of the tetraspanins superfamily of glycoproteins. It was originally identified in prostate carcinoma [22]. Down-regulated expression of CD82 was found in most metastatic cancers [23]. The latest studies have indicated that aberrant expression of CD82 could be a useful marker for metastatic and prognostic factor in many tumors. The relationship among CSC, angiogenesis, and aberrant expression of suppressor of metastasis of tumor in infiltrating ductal carcinoma (IDC) has not yet been explored. In this research, we performed an immunohistochemical investigation to explore the role of the expression of CD133, CD44, CD82, and MVD in clinicopathology and prognosis in 325 specimens of IDC patients.

## Methods

### Patients and specimens

Paraffin-embedded sections of 325 IDC and their correspondent adjacent tissues were obtained from the Department of Pathology, the First Affiliated Hospital of Bengbu Medical College from January 2004 to December 2008. We excluded patients who received preoperative chemotherapy or radiotherapy or other anti-tumor therapy. This research was approved by the ethical committee of Bengbu Medical College before its start. The age of the patients ranged from 21 to 74 years, the median age was 45.6 years. There were 91 cases whose tumors diameter were <2.0 cm, 198 cases that were 2.0 to 4.0 cm, and 36 cases that were ≥4.0 cm. Typing of primary tumors was performed according to the WHO classification, while the Ellis and Elston system was used for grading. Seventy-six cases were at grade 1, 159 cases were at grade 2, and 90 cases were at grade 3. One hundred seventy cases were at the left breast and 155 cases were at the right breast. A total of 155 cases had no lymph node metastasis, whereas 170 cases showed lymph node metastasis. According to clinical staging of pathologic tumor-node-metastasis (pTNM), 54 cases were stage I, 123 cases were stage II, 141 cases were stage III, and 7 cases were stage IV.

### Immunohistochemical analysis

All samples were fixed with 10% buffered formalin and embedded in paraffin. Four-micrometer-thick tissue sections were used for the experiment. All sections were deparaffinized and dehydrated with xylene and graded alcohol. Then, the sections were washed in phosphate-buffered saline (PBS, pH 7.2) for 10 minutes. The endogenous peroxidase activity was quenched by incubation in methanol containing 3% H_2_O_2_ at room temperature for 10 minutes, then heated at 95°C to repair antigens for 30 minutes, and rinsed in PBS several times. The sections were blocked by goat serum at room temperature for 20 minutes and incubated with mouse monoclonal CD133 (Abcam, Burlingame, CA, USA), CD44 (LabVision, Thermo Fisher Scientific, Waltham, MA, USA), CD34 (LabVision), and CD82 (Santa Cruz, Dallas, TX, USA) primary antibodies overnight at 4°C in a humidified chamber. Replacing the primary antibodies with PBS, performed in the negative control group, the slides were incubated with a polymer enhancer (reagent A) at room temperature for 20 minutes. Washing with PBS, the slides were incubated with goat anti-mouse antibody (reagent B) at room temperature for 30 minutes. After a complete wash by PBS, the slides were develop in freshly prepared diaminobenzidine (DAB) solution for 8 minutes, then counterstained with hematoxylin, dehydrated, air-dried, and mounted.

### Evaluation of score

Slides were reviewed independently by two pathologists to evaluate the results of immunohistochemical staining under the light microscope. Ten high power fields were randomly selected from each slide for scoring expression of CD133, CD44, and CD82 proteins; both the extent and intensity of immunohistochemical staining were considered. The intensity of positivity was scored as follows: negative was 0, weak was 1, moderate was 2, and strong was 3. The extent of positivity was scored according to the percentage of positive staining cells: <10% as 1, 11% to 50% as 2, 51% to 75% as 3, and >75% as 4. The final score was determined by multiplying the intensity of positivity and the extent of positivity scores, which yielded a range score from 0 to 12. Expression of CD133, CD44, and CD82 was considered positive when the scores were ≥3.

The staining for CD133 and CD44 was mainly confined to the cell membrane and cytoplasm. The positive staining for CD82 was mainly confined to the cell membrane and cytoplasm. The positive staining was presented as brown granular materials. The positive staining for CD34 was mainly confined to the cytoplasm and membrane. MVD determined the mean score of small CD34+ vessels counted. The modified Weidner’s method was used to calculate the MVD in IDC [24].

### Statistical analysis

Fisher’s exact test, Pearson chi-square test for trends in proportions, *t* test analysis, Spearman’s correlate analysis, and Kaplan-Meier’s method with log rank test or Cox regression method for univariate or multivariate overall survival analysis were used to assess the associations among the positive staining of CD133 or CD44 or CD82 or MVD and clinicopathological indices by SPSS 17.0 software for windows (Chicago, IL, USA). A value of *P* < 0.05 was considered statistically significant.

## Results

### The relationship between expression of CD133, CD44, CD82, and MVD and clinicopathological factors

**Figure 1:**
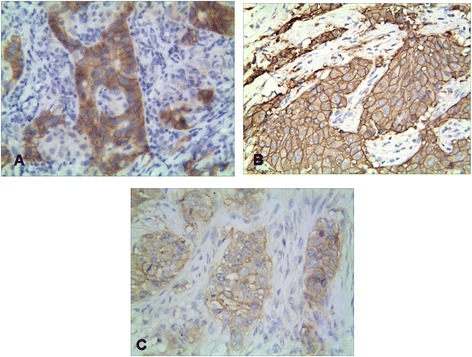
**Expressions of CD133, CD44, and CD82 proteins. (A)** Expression of CD133 protein in infiltrating ductal carcinoma. CD133 was expressed as positive in the membrane and cytoplasm of cancer cells (intensity of positivity: strong = 3, percentage of positive cells: >75% = 4, final score = 12, CD133 × 400, G 2). **(B)** Expression of CD44 protein in IDC. CD44 was expressed as positive in the membrane and cytoplasm of cancer cells (intensity of positivity: strong = 3, percentage of positive cells: >75% = 4, final score = 12, CD44 × 400, G 2). **(C)** Expression of CD82 protein in IDC. CD82 was expressed as positive in the membrane and cytoplasm of cancer cells (intensity of positivity: moderately = 2, percentage of positive cells: >75% = 4, final score = 8, CD82 × 400, G 2).

The positive expression of CD133 and CD44 protein was 48.6% (158/325) and 53.8% (175/150) in the IDC and 9.2% (30/325) and 1.5% (5/325) in the control group, respectively. There was a significant difference between the IDC group and the control group (*P* < 0.001) (Figure 1A, B). And there was a significant difference between the expression of CD133 and CD44 protein and grade of tumors, lymph node metastasis, and pTNM stage (*P* < 0.05). The positive expression of CD82 was 42.2% (137/325) in the IDC group and 98.5% (320/325) in the control group (Figure 1C). There was a significant difference between the IDC group and the control group (*P* < 0.001). And there was a negative relationship between the expression of CD82 protein expression and the grade of tumors, lymph node metastasis, and pTNM stage (*P* < 0.05) (Table 1). The score of MVD of IDC was 20.5 ± 7.0, and the MVD score was found to be closely linked to grade of tumors, lymph node metastasis, and pTNM stage (*P* < 0.05) (Table 1). However, the positive expression of CD133, CD44, and CD82 and the score of MVD had no significant relationship with age, diameter of tumor, and site of tumor (*P* > 0.05) (Table 1).

**Table 1:** **Values of CD133, CD44, and CD82 expression and MVD and clinicopathological characteristics in IDC**

**Variable**	**CD133**		***P*** **value**	**CD44**		***P*** **value**	**CD82**		***P*** **value**	**MVD mean**	***F***	***P*** **value**
	**Negative**	**Positive**		**Negative**	**Positive**		**Negative**	**Positive**				
Age (years)												
<45	73	73	0.652	70	76	0.559	88	58	0.423	22.1 ± 8.0	12.297	0.001
≥45	94	85		80	99		100	79		19.2 ± 5.7		
Site												
Left	90	80	0.557	75	95	0.441	101	69	0.549	20.6 ± 7.1	0.038	0.846
Right	77	78		75	80		87	68		20.4 ± 6.8		
Diameter												
<2.0 cm	54	37	0.197	47	44	0.272	43	48	0.033	21.3 ± 6.5	0.701	0.497
2.0 to 5.0 cm	95	103		90	106		120	78		20.4 ± 6.7		
≥5.0 cm	18	18		13	23		25	11		19.6 ± 9.1		
Grade of tumor												
1	49	27	0.033	45	31	0.019	33	43	0.013	19.8 ± 6.6	3.218	0.041
2	76	83		71	88		101	58		21.2 ± 6.6		
3	42	48		34	56		54	36		20.0 ± 7.9		
Lymph node metastasis												
No	102	53	<0.001	83	72	0.011	74	81	<0.001	18.7 ± 5.6	20.633	<0.001
Yes	65	105		67	103		114	56		22.2 ± 7.7		
TNM stage												
I + II	104	73	0.004	91	86	0.038	93	84	0.034	18.8 ± 6.1	57.775	<0.001
III + IV	63	85		59	89		95	53		22.6 ± 7.3		
ER expression												
Negative	57	70	0.060	46	81	0.004	79	48	0.203	21.8 ± 6.9	13.109	<0.001
Positive	110	88		104	94		109	89		19.8 ± 6.9		
PR expression												
Negative	67	80	0.057	69	78	0.796	78	69	0.112	20.7 ± 7.3	0.195	0.659
Positive	100	78		81	97		110	68		20.4 ± 6.7		
Her-2 expression												
Negative	110	85	0.026	100	95	0.023	102	93	0.013	19.7 ± 6.9	5.397	0.021
Positive	57	73		50	80		86	44		21.9 ± 6.9		

### Prognosis and multivariate analysis

**Figure 2:**
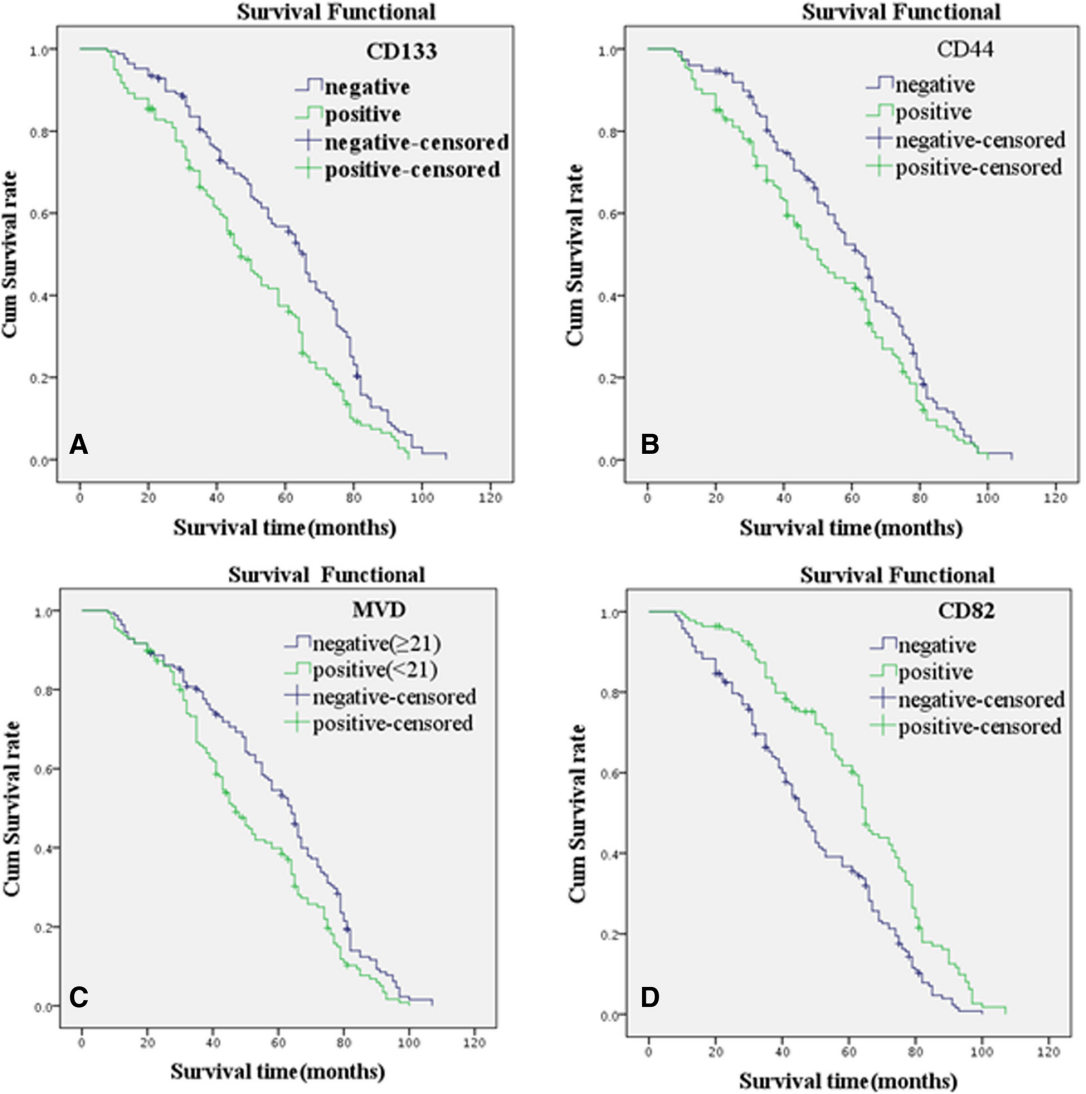
**Kaplan-Meier survival analysis by CD133, CD44, MVD, and CD82 statuses. (A)** Kaplan-Meier survival analysis by CD133 status (*n* = 325). Mean overall survival (OS) time was 46.8 months for the CD133-positive group and 57.9 months for the CD133-negative group (log rank = 16.243, *P* < 0.001). **(B)** Kaplan-Meier survival analysis by CD44 status (*n* = 325). Mean OS time was 48.7 months for the CD44-positive group and 57.0 months for the CD44-negative group (log rank = 5.340, *P* = 0.021). **(C)** Kaplan-Meier survival analysis by MVD status (*n* = 325). Mean OS time was 48.4 months for the MVD ≥21 group and 57.0 months for the MVD <21 group (log rank = 9.623, *P* = 0.002). **(D)** Kaplan-Meier survival analysis by CD82 status (*n* = 325). Mean OS time was 60.8 months for the CD82-positive group and 46.5 months for the CD82-negative group (log rank = 23.644, *P* < 0.001).

Follow-up data showed that there was a significantly decreasing trend in the overall mean survival time between the carcinomas with the positive expression of CD133 protein (46.8 months) and those negative expression of CD133 (57.9 months) (log rank = 16.243, *P* < 0.001, Figure 2A). The survival time of the CD44 positive expression group was significantly less than that of the negative expression group (log rank = 5.340, *P* = 0.021, Figure 2B). The survival time of the score of MVD <21 group was significantly longer than that of the score ≥21 group (log rank = 9.623, *P* = 0.002, Figure 2C). The survival time of the CD82-positive expression group was significantly longer than that of the negative expression group (log rank = 23.644, *P* < 0.001, Figure 2D). A multivariate analysis revealed that the expression of CD133, CD82, and PR and pTNM stage was independent prognosis factors for overall survival time of IDC (Table 2).

**Table 2:** **Multivariate survival analysis of 325 patients with IDC**

**Covariable**	***B***	**SE**	***P***	**RR**	**95% CI**
CD133	0.328	0.135	0.015	1.388	1.065 to 1.809
CD82	−0.484	0.147	0.001	0.617	0.462 to 0.823
PR	−0.376	0.149	0.011	0.687	0.513 to 0.919
pTNM	0.290	0.146	0.047	1.336	1.004 to 1.777

### Correlation of CD133, CD44, CD82, and the score of MVD in IDC

In the positive expression of CD133 group, the mean score of MVD was 22.1 ± 6.3; in the negative expression of CD133 group, the mean score of MVD was 19.1 ± 7.3. There was a positive association between CD133 expression and the score of MVD (*r* = 0.270, *P* < 0.001) and the same association between CD44 expression and CD133 expression (*r* = 0.123, *P* = 0.027). There was a negative association between CD82 expression and CD133 expression (*r* = −0.120, *P* = 0.031) or CD44 expression (*r* = −0.372, *P* < 0.001) or the score of MVD (*r* = −0.194, *P* < 0.001) or Her-2 expression (*r* = −0.137, *P* = 0.013) (Table 3).

**Table 3:** **Values of CD133, CD44, and CD82 expression and MVD**

**Variable**	**CD133**		***r***	***P***	**CD44**		***r***	***P***	**CD82**		***r***	***P***
	**Negative**	**Positive**			**Negative**	**Positive**			**Negative**	**Positive**		
MVD												
<22 group	109	59	0.279	<0.001	88	80	0.129	0.020	82	86	−0.189	0.001
≥22 group	58	99			62	95			106	51		
CD44												
Negative	87	63	0.123	0.027					57	93	−0.372	<0.001
Positive	80	95							131	44		
CD82												
Negative	87	101	−0.120	0.031	57	131	−0.372	<0.001				
Positive	80	57			93	44						

## Discussion

It has been well accepted that CSC plays an important role in tumorigenesis and tumor development. The small population of CSC has been found in many solid tumors including breast cancer (BC) and was related to the poor clinical prognosis [25-27]. The aim of this study was to investigate the expression of the CSC markers CD133, CD44, and CD82 and the score of MVD in IDC and find suitable molecular targets for therapy and predict the prognosis of IDC patients.

CD133 is the most common marker of CSC and has been used as a marker to identify CSC in many solid tumors. In this research, we used immunohistochemical staining to detect the CD133 protein expression in 158 (48.6%), and it was significantly related to the poor prognosis of IDC patients. Further study showed that CD133 protein expression was correlated with grade of tumor, TNM stage, and lymph node metastasis. In addition, we documented a poor survival time with positivity of CD133 protein expression. This research was consistent with previous researches [16,28,29]. However, there was often a controversy whether CD133 was a marker of CSC or not. Some studies [16,30,31] had showed that the expression of CD133 was not only in CSC but also in normal tissues. Some studies thought that CD133 might play a critical role in tumorigenesis [16,30]. Although this study showed that the expression of CD133 was broadly distributed in IDC cells, only a minor part of cells which CD133 was positive expression possess the capacity of stem cells [16,32]. And these cells after routine chemotherapy or radiotherapy may lead to tumor recurrence and metastasis [33].

CD44 has also been used as a marker of CSC. CD44-positive population was found to be more efficient at proliferating and forming clones than CD44 negative tumor cells [34]. In this study, we found the association of increased CD44 protein expression with aggressive tumor-related features, including advanced stage, grade, and lymph node metastasis. We also found a worse survival time with positive expression of CD44 protein in univariate, but not multivariable survival time analysis. This result was consistent with previous studies in other tumor types [35,36].

MVD is the most common standard of measuring tumor angiogenesis and is closely linked to tumor growth and prognostic of patients. MVD may represent the differentiation and vascular network parameters in tumors [35]. In our research, we confirm the relationship between the increased score of MVD with advanced stage, grade, and lymph node metastasis and a shorter survival time. A similar result was found in other studies [16,37].

CD82 is a gene which is located on human chromosome 11p11.2 and plays a critical role in cell adhesion, migration, signaling, and invasion [38,39]. In our research, we found that CD82 protein expression was negatively linked to the advanced stage, grade, and lymph node metastasis. Further survival analysis showed that the patients with CD82-positive expression had a significantly longer survival time than that of CD82-negative expression. Loss-expression or down-expression of CD82 promotes tumor cell invasion and metastasis. CD82 can inhibit tyrosine phosphorylation of β-catenin and stabilize E-cadherin-β-catenin complexes and, thus, prevent tumor cell dissemination from primary tumors [40,41].

Furthermore, CD82 expression was negatively associated with CD133 and CD44 expression and MVD. And there was a positive relationship between CD133 expression or CD44 expression or MVD. Only a small part of tumor cells which expressed CD133 protein possessed the capacity of CSC. The niche where CSC resides can regulate CSC self-renewal, so vascular niche or neovascularization can regulate CSC fate [16]. CSC can promote tumor cell proliferation, and tumors require blood and nutrition for growth, invasion, and metastasis. These tumor cells can stimulate angiogenesis in order to get adequate blood and nutrition. CSC may contribute to the formation of neovascularization [21]. CSC can meet their need to manipulate stromal cells by inducing a premetastatic niche in distant organs for their arrival [42]. Loss-expression or down-expression of CD82 can further promote the metastatic ability of tumor cells.

## Conclusions

It is suggested that CSC may play a critical role in the evolution of IDC. The combined detection of CD133, CD44, CD82, and MVD, to some extent, may reflect the biological behavior and be considered as potential markers for the prognosis of IDC, thus give the choice of molecular target therapy.
